# Circular RNA alterations are involved in resistance to avian leukosis virus subgroup-J-induced tumor formation in chickens

**DOI:** 10.18632/oncotarget.16442

**Published:** 2017-03-22

**Authors:** Xinheng Zhang, Yiming Yan, Xiaoya Lei, Aijun Li, Huanmin Zhang, Zhenkai Dai, Xinjian Li, Weiguo Chen, Wencheng Lin, Feng Chen, Jingyun Ma, Qingmei Xie

**Affiliations:** ^1^ College of Animal Science, South China Agricultural University, Guangzhou, 510642, P.R. China; ^2^ Guangdong Provincial Key Lab of Agro-Animal Genomics and Molecular Breeding, Guangzhou, 510642, P.R. China; ^3^ College of Science and Engineering, Jinan University, Guangzhou, 510632, P.R. China; ^4^ USDA, Agriculture Research Service, Avian Disease and Oncology Laboratory, East Lansing, MI, 48823, USA; ^5^ Key Laboratory of Animal Health Aquaculture and Environmental Control, Guangdong, Guangzhou, 510642, P.R. China; ^6^ South China Collaborative Innovation Center for Poultry Disease Control and Product Safety, Guangzhou, 510642, P.R. China

**Keywords:** circular RNA, ALV-J-resistant chickens, circular-seq, tumor

## Abstract

Avian leukosis virus subgroup (ALV-J) is an oncogenic neoplasm-inducing retrovirus that causes significant economic losses in the poultry industry. Recent studies have demonstrated circular RNAs (circRNAs) are implicated in pathogenic processes; however, no research has indicated circRNAs are involved in resistance to disease. In this study, over 1800 circRNAs were detected by circRNA sequencing of liver tissues from ALV-J-resistant (*n* = 3) and ALV-J-susceptible chickens (*n* = 3). 32 differentially expressed circRNAs were selected for analyzing including 12 upregulated in ALV-J-resistant chickens and 20 upregulated in ALV-J-susceptible chickens, besides, the top five microRNAs (miRNAs) for 12 upregulated circRNAs in ALV-J-resistant chickens were analyzed. Gene ontology and KEGG pathway analyses were performed for miRNA target genes, the predicted genes were mainly involved in immune pathways. This study provides the first evidence that circRNA alterations are involved in resistance to ALV-J-induced tumor formation. We propose circRNAs may help to mediate tumor induction and development in chickens.

## INTRODUCTION

Circular RNAs (circRNAs) are recently discovered noncoding RNAs that are highly represented in the eukaryotic transcriptome [1]. Previously regarded as splicing error byproducts [2], circRNAs are mainly formed by back-splicing of covalently jointed 3`-and 5`-ends [3]. Abundant expression of circRNAs that compete with endogenous RNAs has been reported [4]. To date, three types have been reported: circular intronic RNA, exonic circRNA and retained-intron circRNA [5–7]. Several functions have been identified for circRNAs, including RNA transport [8], protein binding [9] and regulation of translation [5]. In 2013, circRNAs were identified as efficient microRNA (miRNA) sponges [10, 11]. Endogenous linear RNAs have the ability to sequester and inhibit miRNA activity [12], indicting the existence of close interactions between circRNAs, miRNAs and mRNAs. Moreover, circRNAs have been associated with diseases, including Alzheimer disease, colorectal and ovarian cancer, idiopathic lung fibrosis and hepatocellular carcinoma [13–15].

The oncogenic retrovirus avian leukosis virus (ALV) causes a major infectious disease that leads to poor egg laying performance in chickens and impacts the poultry industry worldwide [16]. There are seven subgroups of ALV in chickens: ALV-A, -B, -C, -D, -E, -J and –K [17, 18]. ALV-J was first reported in commercial meat-type chickens in England in 1988 [19]. ALV infection spreads by horizontal transmission, vertical or congenital transmission and genetic transmission in flocks [20, 21]. Chickens infected with ALV-J can develop immunosuppression together with diverse tumors, such as myelocytomas, sarcomas, hemangiomas, nephromas and erythroblastosis, as well as myeloid leukosis, and the virus induces high mortality [22–24]. Up to now, no effective vaccine has been developed for ALV-J. The control and eradication of ALV-J from pedigree generations has become a priority in the breeder industry [25]. Breeding chickens that are naturally resistant to ALV-J infection is one potential control measure.

In a previous study, we bred ALV-J-resistant and -susceptible chickens and identified an anti-tumor gene by RNA sequencing [17]. To assess whether circRNAs are involved in resistance to ALV-J-induced tumor formation, tissue samples from ALV-J-resistant and -susceptible chickens were subjected to circRNA sequencing. The study lays the foundation of circRNAs may be used as a molecular marker of ALV-J-resistance in chickens and indicates circRNAs could play a role in cancer in chickens.

## RESULTS

### CircRNAs expression profiles

**Figure 1:**
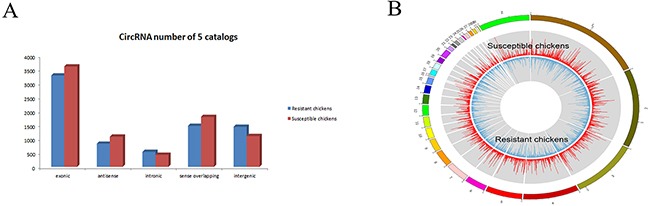
CircRNA sequencing data **(A)** Mapping region distribution within read alignments; **(B)** Circos plots, outline is the reference genome, inside is the chromosome coverage across all samples.

**Figure 2:**
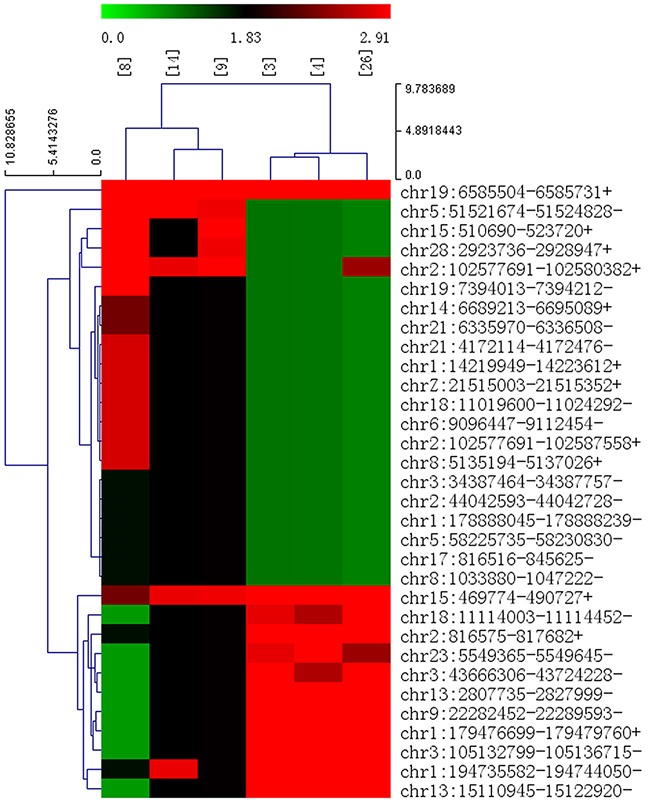
Heap map for the 32 significant differentially expressed circRNAs in ALV-J-resistant chickens Sample numbers [4], [3] and [26] represent ALV-J-resistant chickens; [8], [14] and [9] represent ALV-J-susceptible chickens. The numbers 0.0, 1.83 and 2.91 on the top represent the expression levels of the circRNAs. Each row represents a circRNA and each column represents one sample. Dendrograms produced by clustering analysis of the samples are shown on the top. Twelve circRNAs were upregulated (red) and 20 were downregulated (green) in ALV-J-resistant chickens compared to ALV-J-susceptible chickens.

In total, the numbers of reads from the three liver tissue samples from ALV-J-resistant chickens were 71314622, 69193854 and 71259856, resulting in mapped read counts of 55805408, 55911773 and 56634896 respectively (Figure 1A). The numbers of reads from the three tissue samples from ALV-J-susceptible chickens were 65310898, 64652876, 65505628, resulting in mapped read counts of 52943856, 52223375 and 52764699 respectively. Overall, over 1800 circRNAs were detected by circRNA sequencing; the Circos figure for the circRNAs in ALV-J-resistant and -susceptible chickens is shown in Figure 1B. In total, 32 significantly differentially expressed circRNAs were identified in ALV-J-resistant chickens compared to ALV-J-susceptible chickens. A heat map of the 32 differentially expressed circRNAs was generated to illustrate the distinguishable circRNA expression profiling of the samples (Figure 2); As shown in heat map, the number of 0.0, 1.83 and 2.91 on the top represents each circRNA expression level. The each row represents each circRNA and each column represents one sample. Dendrograms from clustering analysis of the samples are exhibited on the top. The left dendrogram separates the expression profiles of the resistant chickens group from the susceptible chickens group. Hierarchical clustering demonstrated the circRNA sequencing data was reliable.

### Differentially expressed circRNAs in ALV-J-resistant chickens

Of the 32 significantly differentially expressed circRNAs, 12 circRNAs were upregulated and 20 were downregulated in ALV-J-resistant chickens compared to ALV-J-susceptible chickens. The 12 upregulated circRNAs in ALV-J-resistant chickens were ranked by fold change (FC), and the names of the best predicted transcript (parent gene) for each of these 12 circRNAs are shown in Table 1.

**Table 1:** The twelve significantly upregulated circRNAs linked to resistance to ALV-J induced tumor formation in chickens

CircRNA ID	*P*-value	Fold change	chrom	txStart	txEnd riginal	Original Gene
chr13:15110945-15122920-	0.0025	5.8790	chr13	15110944	15122920	JADE2
chr9:22282452-22289593-	0.0050	4.2395	chr9	22282451	22289593	LEKR1
chr19:6585504-6585731+	0.0062	4.0622	chr19	6585503	6585731	UbI
chr3:105132799-105136715-	0.0297	3.8360	chr3	105132798	105136715	NCOA1
chr1:179476699-179479760+	0.0114	3.4851	chr1	179476698	179479760	KDELC2
chr15:469774-490727+	0.0166	3.2521	chr15	469773	490727	PPM1F
chr1:194735582-194744050-	0.0291	3.1591	chr1	194735581	194744050	RAB6A
chr18:11114003-11114452-	0.0367	2.9598	chr18	11114002	11114452	CASKIN2
chr13:2807735-2827999-	0.0156	2.8263	chr13	2807734	2827999	RANBP17
chr3:43666306-43724228-	0.0265	2.7662	chr3	43666305	43724228	PACRG
chr23:5549365-5549645-	0.0334	2.6458	chr23	5549364	5549645	HMGCL
chr2:816575-817682+	0.0031	2.5679	chr2	816574	817682	SMARCC1

### The function analysis of miRNA target genes

**Figure 3:**
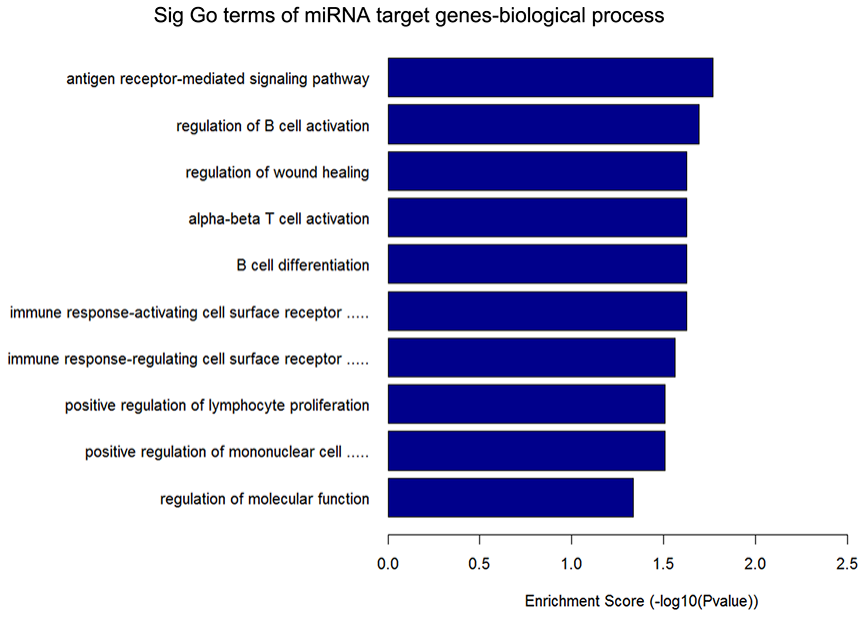
Annotated significant pathways targeted by the miRNA target genes of 12 circRNAs upregulated in ALV-J-resistant chickens The horizontal axis is the –LgP (logarithm of *P*-value) for the pathway and the vertical axis is the pathway category. *P* < 0.05 was considered significant.

**Figure 4:**
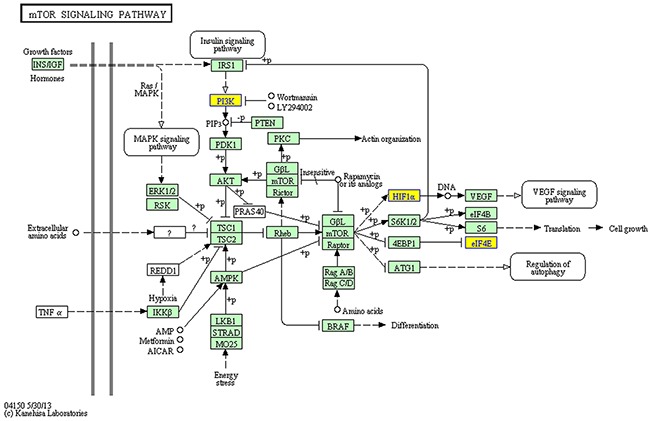
KEGG pathway analysis for the miRNA target genes of 12 circRNAs upregulated in ALV-J-resistant chickens The yellow boxes indicate the genes targeted by differentially expressed circRNAs.

GO annotation of the miRNA target genes predicted to be targeted by the 12 circRNAs that were upregulated in ALV-J-resistant chickens revealed the target genes were mainly involved in immune pathways (Figure 3), such as regulation of B cell activation, the immune response-activating cell surface receptor signaling pathway, B cell differentiation, alpha-beta T cell activation and positive regulation of mononuclear cell proliferation. KEGG pathway analysis of the predicted miRNA target genes is shown in Figure 4, three of the predicted miRNA target genes are associated with the mTOR signaling pathway.

### Validation of circRNA expression by qRT-PCR

**Figure 5:**
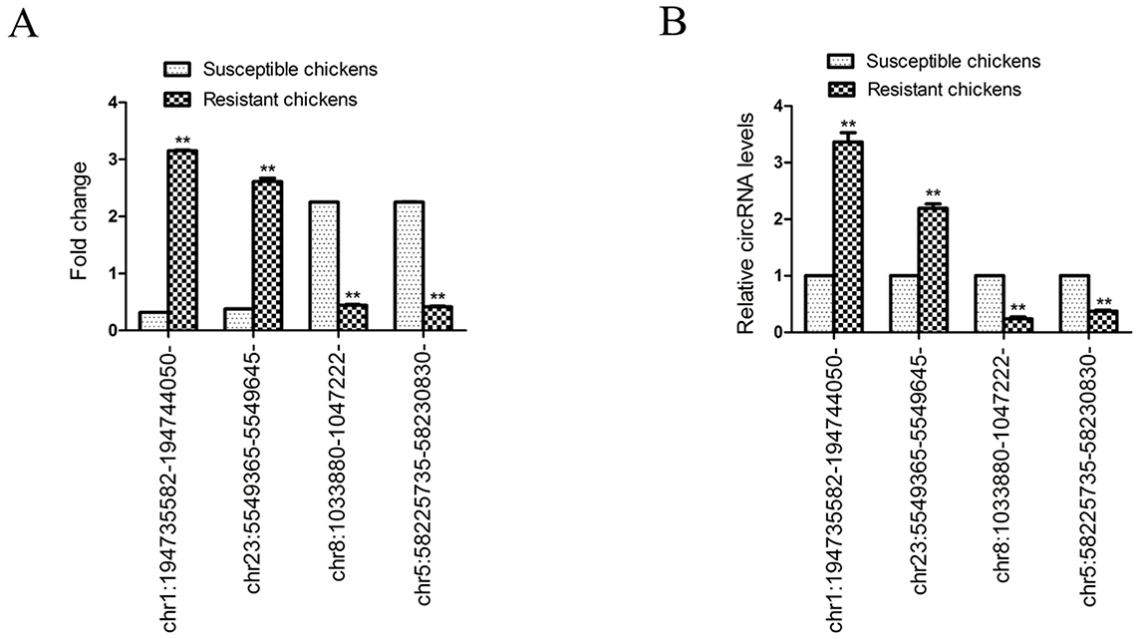
Validation of the expression of four selected differentially expressed circRNAs identified by circRNA sequencing **(A)** Fold change for each circular RNA as indicated by circRNA sequencing; **(B)** Relative circRNA levels as indicated by qPCR. Bars are mean ± SD (n=3); **P < 0.05.

Two randomly selected circRNAs that were upregulated in ALV-J-resistant chickens (chr1:194735582-194744050- and chr23:5549365-5549645-) and two randomly circRNAs that were downregulated in ALV-J-resistant chickens (chr8:1033880-1047222- and chr5:58225735-58230830-) were selected to validate the expression levels of the significantly differentially expressed circRNAs by quantitative real-time polymerase chain reaction (qRT-PCR). The qRT-PCR data for the four circRNAs was consistent with the trends observed using circRNA sequencing (Figure 5).

### miRNA binding sites prediction and circRNA-targeted miRNA-gene network

**Figure 6:**
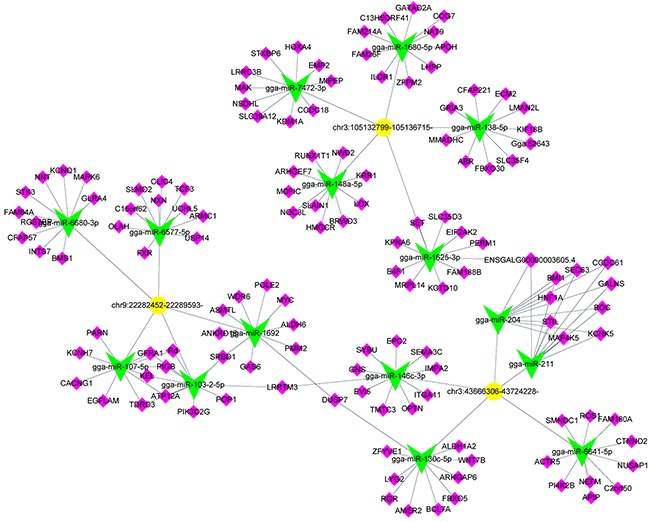
Predicted biomathematical circRNA-miRNA-gene network for three selected upregulated circRNAs in ALV-J-resistant chickens Yellow represents circRNAs, green represents miRNAs and pink represents miRNA target genes.

CircRNAs with high number of target sites for a specific miRNA, the top 5 predicted miRNA targets of each circRNA were listed in Table 2. We selected 3 differentially up-regulated circRNAs (chr3:43666306-43724228-, chr9:22282452-22289593- and chr3:105132799-105136715-) and constructed the CircRNA-targeted miRNA-gene network. Each circRNA had 5 predicted miRNA targets, every miRNA target had 10 predicted genes (Figure 6). The network showed that circRNA-targeted miRNA-gene had a strong connection with each other.

**Table 2:** Predicted miRNAs for 12 differentially expressed circRNAs linked to resistance to ALV-J induced tumor formation in chickens

CircRNA ID	miRNA	miRNA	miRNA	miRNA	miRNA
chr13:15110945-15122920-	gga-miR-6634-5p	gga-miR-1649-5p	gga-miR-24-3p	gga-miR-6587-3p	gga-miR-1663-3p
chr9:22282452-22289593-	gga-miR-103-2-5p	gga-miR-107-5p	gga-miR-6680-3p	gga-miR-1692	gga-miR-6577-5p
chr19:6585504-6585731+	gga-miR-3530-3p	gga-miR-6549-5p	gga-miR-1664-5p	gga-miR-1764-5p	gga-miR-193a-5p
chr3:105132799-105136715-	gga-miR-7472-3p	gga-miR-138-5p	gga-miR-1625-3p	gga-miR-148a-5p	gga-miR-1680-5p
chr1:179476699-179479760+	gga-miR-6632-5p	gga-miR-153-5p	gga-miR-1705	gga-miR-1684b-3p	gga-miR-6698-3p
chr15:469774-490727+	gga-miR-2129	gga-miR-1632-5p	gga-miR-1659	gga-miR-6698-3p	gga-miR-6598-5p
chr1:194735582-194744050-	gga-miR-6660-3p	gga-miR-1662	gga-miR-15c-5p	gga-miR-15b-5p	gga-miR-1787
chr18:11114003-11114452-	gga-miR-6607-5p	gga-miR-1749-3p	gga-miR-1785	gga-miR-19b-5p	gga-miR-6545-3p
chr13:2807735-2827999-	gga-miR-365b-5p	gga-miR-1756b	gga-miR-217-3p	gga-miR-6690-5p	gga-miR-1771
chr3:43666306-43724228-	gga-miR-146c-3p	gga-miR-130c-5p	gga-miR-211	gga-miR-204	gga-miR-6641-5p
chr23:5549365-5549645-	gga-miR-6702-5p	gga-miR-1662	gga-miR-34b-5p	gga-miR-6611-5p	gga-miR-6641-5p
chr2:816575-817682+	gga-miR-6574-5p	gga-miR-6577-5p	gga-miR-1596-5p	gga-miR-1786	gga-miR-1615

## DISCUSSION

ALV-J is a major pathogen with diverse pathotypes that leads to enormous economic losses. ALV-J has rapidly evolved with respect to its pathogenicity, host range and range of tumors induced [26]. ALV-J-induced tumors among layer-type chickens are one of the most important problems facing the global poultry industry [27]. Although the tumorigenic and pathogenic mechanisms of ALV-J remain unclear and are hot topics of research, no vaccine has been developed to control this disease; therefore, assessment of the host to identify genes associated with resistance to ALV-J-induced tumors may provide a novel method of controlling this problem. In this respect, we previously bred ALV-J-resistant and -susceptible chickens and discovered an anti-tumor gene by RNA sequencing [17].

CircRNAs are a recently discovered type of noncoding RNA that have attracted significant attention [28]. Numerous studies have identified hundreds and thousands of different circRNAs, mostly comprising one to several exons of protein coding genes [14], and circRNAs have been shown to regulate gene expression in mammals [9]. A recent publication indicated that circular RNA was enriched and stable in exosomes and could be a promising biomarker for cancer diagnosis [29], besides, Circular RNA expression alterations were also involved in disease occur and the detailed analysis methods of circRNA alterations were also illustrated [30]. MiRNAs are highly conversed during evolution and negatively regulate target mRNAs. Moreover, a previous study demonstrated that miRNAs play critical roles in tumor formation in chickens infected with ALV-J [31]. However, there is no data on the role of circRNAs in resistance to tumor formation induced by ALV-J or other pathogens in any species. In the current study, we assessed whether circRNAs are involved in resistance to ALV-J-induced tumor formation by analyzing circRNA expression in ALV-J-resistant and -susceptible chickens [17] using circRNA sequencing.

Gene ontology (GO) is a widely used bioinformatics concept that unifies the genes and gene products of all species [32]. High-throughput circRNA sequencing and real-time PCR are standardized approaches used to assess circRNA expression; these methods have been widely used to infer host gene expression in response to disease [33, 34]. Our study demonstrates circRNAs are differentially expressed in ALV-J-resistant chickens, including the 12 upregulated circRNAs, indicating circRNAs are involved in resistance to ALV-J-induced tumor formation. In 2011, an endogenous circRNA was found to be highly expressed in human and mouse brain tissues [35], but the function of this circRNA remained unknown. In 2013, circRNAs were identified as efficient microRNA (miRNA) sponges [10]. Overexpressing miRNA target site concatamers (miRNA sponges) results in loss of miRNA function accompanied by increased endogenous target gene expression [11]. Considering that circRNA can absorb miRNA and Go terms of miRNA target genes of 12 differentially expressed circRNAs that were upregulated in ALV-J-resistant chickens in this study are linked to immune pathways such as antigen receptor signaling pathway and regulation of B cell activation, besides, three of the predicted miRNA target genes in KEGG pathway are associated with the mTOR signaling pathway which is a key regulator of cell growth and proliferation and mTOR dysregulation has a key role to play in various cancers [36], so we suspect circRNAs could be involved in initiation of the immune effects that protect ALV-J-resistant chickens from infection and tumor formation. We propose circRNAs may represent a new mediator of tumor initiation and progression.

Further study of the functions of these circRNAs could improve our understanding of the mechanisms by which circRNAs confer resistance against ALV-J-induced tumor formation. This data also provides a basis for assessing the potential of the differentially expressed circRNAs as novel of biomarkers of resistance to ALV-J-induced tumor formation in chickens. Moreover, this study provides a solid theoretical foundation for further research into the molecular mechanisms by which circular RNAs play a role in the initiation and progression of cancer in chicken.

## MATERIALS AND METHODS

### Ethics statement

All experiments were carried out in strict accordance with the recommendations of the Guide for the Care and Use of Laboratory Animals of the National Institutes of Health. The use of animals in this study was approved by South China Agricultural University Committee of Animal Experiments (approval ID: 201004152).

### Samples and RNA isolation

ALV-J-resistant and -susceptible chickens (F3 generation) were used as classical models of resistance or easily-induced tumors in response to ALV-J challenge [17]. The standard ALV-J NX0101 strain which induced myeloid tumors was used in this study. RNA was isolated from livers of three ALV-J-resistant and three ALV-J-susceptible chickens at 20 weeks after challenge with ALV-J. Total RNA was isolated from each group using TRizol reagent (Life Technologies, Carlsbad, CA, USA) according to the manufacturer's instructions. The concentration of the RNA samples was determined by assessing the OD260/280 using a NanoDrop ND-2000 instrument (Thermo, Waltham, MA, USA). The integrity of the RNA samples was assessed by denaturing agarose gel electrophoresis.

### RNA library construction and circRNA sequencing

Total RNA from each sample was used to prepare the circRNA sequencing library, via the following steps: 1) 5 μg total RNA samples were pretreated to enrich circRNAs using the CircRNA Enrichment Kit (Cloud-seq Inc., Shanghai, China). RNA libraries were constructed from the treated RNAs using the TruSeq Stranded Total RNA Library Prep Kit (Illumina, San Diego, CA, USA) according to the manufacturer's instructions. Libraries were controlled for quality and quantified using the BioAnalyzer 2100 system (Agilent Technologies, Inc., Santa Clara, CA, USA). The libraries were denatured as single-stranded DNA molecules, captured on Illumina flow cells, amplified *in situ* as clusters and finally sequenced for 150 cycles on an Illumina HiSeq Sequencer according to the manufacturer's instructions.

### CircRNA sequencing analysis

Paired-end reads were harvested from the Illumina HiSeq 4000 sequencer and quality controlled using Q30 [37]. After 3’ adaptor-trimming and removal of low quality reads using Cutadapt software [38], the high quality trimmed reads were used for analysis of circRNAs. The high quality reads were aligned to the reference genome/transcriptome using bowtie2 software and circRNAs were detected and identified using find_circ software [39, 1]. Raw junction reads for all samples were normalized to the number of total mapped reads and log2 transformed. Circos software was used to construct the circos figure [40]. CircRNAs exhibiting fold changes ≥ 2.0 with *P*-values ≤ 0.05 were classified as significantly differentially expressed circRNAs.

### Bioinformatic analysis and target prediction

CircRNA and miRNA interactions were predicted using customized Arraystar miRNA target prediction software based on TargetScan and miRanda [41, 42]; the top five putative target miRNAs were identified for upregulated circRNAs in ALV-J-resistant chickens. The putative target genes of these miRNAs were identified using Targetscan [41]. Cytoscape software was used to construct the circRNA-miRNA-gene networks [43]. Gene ontology (GO) and Kyoto Encyclopedia of Genes and Genomes (KEGG) analysis were performed for the differentially expressed circRNA-associated genes [44, 45].

### Quantitative real-time polymerase chain reaction analysis

Total RNA was reverse transcribed to synthesize cDNA using PrimeScript RT Reagent Kit (Perfect Real Time; TaKaRa, Osaka, Japan) and subjected to quantitative real-time polymerase chain reaction (qRT-PCR) analysis on an Applied Biosystems 7500 Fast Real-Time PCR System (Roche, Basel, Switzerland; software version 2.0.5) with SYBR Green qPCR SMix (ROX; Roche). The primers used for the qRT-PCR analysis were: chr1:194735582-194744050- (F 5’-GGG CTTGTTTCTGGTTTGGGTTAG-3’, R 5`-AGTGTCCAT TGAGGAAGGAGAAAG-3`), chr23:5549365-5549645- (F 5’-CTCCCTGTCTCCTCTCCCAATCTT-3’, R 5’-CTC TGGTTCCTCTGTACTGCTGTATCC-3’), chr8:1033880- 1047222- (F 5’-GTTCCCCTAAGAAGCATTTGACAGA CT-3’, R 5’-GGGATTAGAAGGAACTCCAGACACG -3’), chr5:58225735-58230830- (F 5’-CTTCAGGGCTT CATCTGCTCTC-3’, R 5’-GACTCCATTACTCCAGAC AAAGTACG-3’), and β-actin mRNA (F 5’-GAAGT ACCCCATTGAACACGG-3’, R 5’-AGGCATACAGGGA CAGCACA-3’). Three independent samples were analyzed for each group; all samples were assessed in triplicate. The 2^−ΔΔCt^ method was used to analyze the qPCR results [46].

### Statistical analysis

Data are expressed as mean ± standard deviation (SD). The Student's *t-*test was used to assess differences among groups; *P* < 0.05 was considered significant.
